# The current landscape of pre-exposure prophylaxis service delivery models for HIV prevention: a scoping review

**DOI:** 10.1186/s12913-020-05568-w

**Published:** 2020-07-31

**Authors:** Jef Vanhamel, Anke Rotsaert, Thijs Reyniers, Christiana Nöstlinger, Marie Laga, Ella Van Landeghem, Bea Vuylsteke

**Affiliations:** Department of Public Health, Institute of Tropical Medicine, Nationalestraat 155, B-2000 Antwerp, Belgium

**Keywords:** HIV prevention, PrEP, Health care providers, Delivery of health care, Sexual health, MSM

## Abstract

**Background:**

Strengthening HIV prevention is imperative given the continued high HIV incidence worldwide. The introduction of oral PrEP as a new biomedical HIV prevention tool can be a potential game changer because of its high clinical efficacy and the feasibility of its provision to different key populations. Documenting the existing experience with PrEP service delivery in a variety of real-world settings will inform how its uptake and usage can be maximised.

**Methods:**

We conducted a scoping review using the five-step framework provided by Arksey and O’Malley. We systematically searched the existing peer-reviewed international and grey literature describing the implementation of real-world PrEP service delivery models reporting on four key components: the target population of PrEP services, the setting where PrEP was delivered, PrEP providers’ professionalisation and PrEP delivery channels. We restricted our search to English language articles. No geographical or time restrictions were set.

**Results:**

This review included 33 articles for charting and analysing of the results. The identified service delivery models showed that PrEP services mainly targeted people at high risk of HIV acquisition, with some models targeting specific key populations, mainly men who have sex with men. PrEP was often delivered centralised and in a clinical or hospital setting. Yet also community-based as well as home-based PrEP delivery models were reported. Providers of PrEP were mainly clinically trained health professionals, but in some rare cases community workers and lay providers also delivered PrEP. In general, in-person visits were used to deliver PrEP. More innovative digital options using mHealth and telemedicine approaches to deliver specific parts of PrEP services are currently being applied in a minority of the service delivery models in mainly high-resource settings.

**Conclusions:**

A range of possible combinations was found between all four components of PrEP service delivery models. This reflects differentiation of care according to different contextual settings. More research is needed on how integration of services in these contexts could be expanded and optimised to respond to key populations with unmet HIV prevention needs in different settings.

## Background

Scaling-up antiretroviral therapy (ART) delivery has saved millions of lives worldwide over the past two decades, both as an effective treatment and as a prevention strategy [1]. Reaching viral suppression with current ART regimens not only drastically improves the health outcomes of people living with HIV, it also eliminates onward transmission towards their sexual partners. Yet despite a 16% reduction in the number of new HIV infections between 2010 and 2018, there were still about 1.7 million new HIV diagnoses worldwide in 2018 [2]. This leaves us far off-track from the pre-set target of 500,000 new infections by 2020, set by UNAIDS within the overall fast-track strategy of ending AIDS as a public health threat by 2030 [3]. Therefore there is a need for a continued focus on combination prevention, i.e. the introduction of multicomponent packages of evidence-based biomedical, behavioral, and structural interventions operating on multiple levels (individual, dyadic, community and societal) [4].

Oral pre-exposure prophylaxis (PrEP) is a promising addition to the HIV prevention toolkit, with its efficacy proven in multiple clinical trials, in different populations and geographical areas [5]. Multiple demonstration studies worldwide have shown the feasibility and acceptability of delivering PrEP to different key populations, such as men who have sex with men (MSM), transgender women (TGW), and adolescent girls and young women (AGYW) [6]. Considering this broad evidence base, the number of countries implementing PrEP has been increasing over the past few years [6–8]. By June 2018, a total of 19 high-income countries (HICs) and 21 low- and middle-income countries (LMICs) had adopted, or had a pending policy for, formal PrEP implementation [7].

Translating the efficacy of early PrEP trials into population-level effectiveness poses implementation challenges. The World Health Organization (WHO) has created an implementation tool to guide health care professionals and policymakers in this quest, describing an effective PrEP program as one in which “*people at substantial risk of HIV are properly identified, offered PrEP and then use PrEP as directed*” [9]. This implies following a cascade of care, described as the PrEP care continuum by Nunn et al. and others [10, 11]. Impacting on HIV incidence will ultimately require addressing all steps in the care cascade as illustrated in the PrEP care continuum [11]. The first promising indications of such population-level effectiveness of large-scale PrEP implementation have been reported by HIV prevention programs, e.g. in London, San Francisco and Australia [12–14]. In these contexts, PrEP as part of a comprehensive HIV prevention package has significantly reduced the number of annual new HIV diagnoses. In some other contexts, the actual relationship between PrEP coverage and annual new HIV diagnoses did not prove to be a straightforward one [15]. To understand why differences in PrEP effectiveness on a population-level may exist, we need better knowledge on how PrEP is being prescribed and provided, and how it is integrated into a combination prevention approach. Insights into the potential variety of ways to deliver PrEP can help in identifying opportunities to optimise PrEP provision outside a research context. This will be needed to maximise its uptake and acceptability, and to support adherence [16]. After all, programs for PrEP need to operate within existing health systems and contexts, addressing very specific populations based on local HIV epidemics. Therefore, there is no ‘one-size-fits-all’ in real-world PrEP implementation.

The objective of this scoping review was to explore and map the literature describing PrEP service delivery models applied in real-world settings. For this purpose, we defined a ‘PrEP service delivery model’ as consisting of four key components: the population targeted for PrEP, the infrastructural setting of PrEP provision, PrEP providers, and the applied delivery channels to make PrEP available. Existing literature reviews on earlier steps in the PrEP care continuum such as assessing eligibility and awareness about PrEP, and barriers and facilitators towards its uptake, do not fully respond to this need [17, 18]. This review therefore focused on the delivery of services to people either initiating PrEP or continuing its use, and on understanding how these systems operate. These insights can assist in identifying possible ways to organise the provision of PrEP and help regional and national programs tailoring PrEP services to best fit their specific population needs, organizational structures and contextual settings. Considering the novelty of the research field and the exploratory nature of the research objective, a scoping review method was used to identify and summarise literature on PrEP (implementation) research over the last few years [19].

## Methods

This scoping review was inspired by the principles of the framework for conducting scoping studies by Arksey & O’Malley and based on the PRISMA extension guidelines for scoping reviews (see Additional file 7) [20, 21]. It was guided by five steps: (1) identifying the research question, (2) identifying relevant studies, (3) study selection, (4) charting the data and (5) collating, summarising and reporting the results.

### Identifying the research question (step 1)

**Fig. 1:**
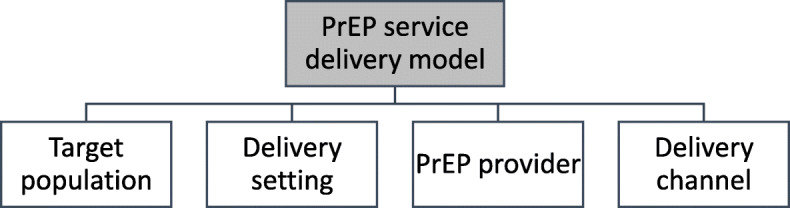
Understanding of the concept ‘PrEP service delivery model’ in this review. Four key components are required, namely the target population, the delivery setting, the PrEP provider and the used delivery channels

To our knowledge, standard definitions of PrEP service provision or delivery are lacking. Guided by the PrEP care continuum as described by Nunn et al. and others, we focused our review on the steps in the continuum where PrEP is being prescribed and provided to people and how care is currently organised around this [10]. The lens we applied to look at this subject is an operational one, as we aimed at making an inventory of practical ‘service delivery models’ currently being rolled-out to provide PrEP care. For the purpose of this review, we defined a ‘PrEP service delivery model’ as consisting of four key components: the organisation of care for individuals who want to initiate and maintain PrEP use (target population) (1), the infrastructure and practical setting of PrEP provision (the delivery setting) (2), type and training of the care providers (PrEP provider) (3) and the platforms or media that were used to deliver PrEP services to the people (delivery channels) (4) (Fig. 1).

In line with this scope, we phrased the review question as follows:

“Which service delivery models for PrEP are currently applied in real-world settings globally, and what are differences and similarities in terms of their target population, the infrastructural setting of PrEP provision, PrEP providers, and delivery channels?”

### Identifying relevant studies (step 2)

We developed a search string using the PubMed search builder (Table 1).

**Table 1:** Construction of the search string in PubMed

Core Concept	Combined search terms
HIV	“HIV”[MeSH] OR “HIV”[tiab] OR “human immunodeficiency virus”[tiab]
PrEP	“Pre-Exposure Prophylaxis”[MeSH] OR “pre-exposure prophylaxis”[tiab] OR “preexposure prophylaxis”[tiab] OR “prep”[tiab]
Health services delivery	“Delivery of Health Care”[MeSH] OR “Drug Delivery Systems”[MeSH] OR “health service provision”[tiab]

Where available, appropriate MeSH terms were used, supported by free-text formats. After having conducted an initial exploratory search, the final search formula consisted of the core concepts (HIV) AND (Pre-Exposure Prophylaxis) AND (Delivery of Health Services). This search formula was subsequently adapted to fit the search strategy for the different databases that were searched (see Additional file 1). This search strategy was not registered in any protocol database. We finally ran the search syntax in the following peer-reviewed electronic databases: MEDLINE (PubMed), Web of Science Core Collection and Google Scholar (to also include grey literature). No time restrictions were set. Monthly e-mail updates were set for the searches in these three databases in order to include articles after the initial moment the databases were searched, up to 1/1/2020.

Additionally, we performed hand-searching of conference archives for relevant abstracts of CROI 2017–2019, AIDS 2018, AIDS IMPACT 2019 and IAS 2019. Relevant abstracts were only included if the full-text had become available by the time of conducting this literature search. Authors were contacted if the full-text could not be found or accessed online.

We also scanned government websites for relevant (national) implementation/strategic documents for PrEP delivery for countries with more than 500 PrEP initiations according to *PrEPWatch.org* (accessed 10/08/2019). Finally, we included articles through snowballing reference lists of primary resources until a point of saturation was reached.

For all articles identified, we extracted DOI-number, year of publication, authors, title and abstract to a spreadsheet program (MS Excel version 1908) using EndNote (version X9.2) as a reference manager.

### Study selection (step 3)

We included full-text articles, reports and book chapters fitting the following inclusion criteria:

English-language, peer-reviewed papers and grey literature with a focus on oral PrEP for HIV prevention and describing health services organisation aspects of PrEP care. In line with the review question, articles should be reporting or investigating ‘models of service delivery’ or providing a framework for organisation of PrEP delivery in the local health system. Articles should therefore focus on stages of the PrEP care continuum related to PrEP provision and retention in care, in a real-world setting, with reporting on four key components: target population, delivery setting, PrEP providers, and delivery channels (see Additional file 2).

We excluded studies focusing on methods of drug administration other than oral (e.g. injections, vaginal or rectal PrEP). Studies focusing on barriers for uptake of PrEP services in general (attitudes/awareness studies) were equally excluded. We included reviews, viewpoints, comments, perspective articles and demonstration studies only if the PrEP service delivery in itself was the main focus, or if they discussed a specific model of PrEP delivery. No geographical restrictions were set.

We iterated the development and application of these criteria within the review team to further improve the study selection. Finally, the pre-defined criteria were applied and tested against a title-and-abstract (tiab) screening for all articles identified through the search strategy described above by one reviewer (JV). A second reviewer (AR) independently assessed a random selection of 10% of the extracted titles and abstracts obtained using the RAND-function in MS Excel. To overcome inter-rater variability, we used the Cohen’s Kappa coefficient, which showed good agreement between the selection decisions of both reviewers (see Additional file 3). We resolved inconsistencies in tiab-selection through discussion among the reviewers.

### Charting the data and collating, summarizing and reporting of the results (step 4 and 5)

We charted the data (step 4) based upon a data extraction sheet that we developed and used to systematically collect data for each included article (see Additional file 4), as per scoping review methodologies [16, 17]. This was done by one reviewer (JV). The categories of acquired data in the extraction sheet formed the basis for the development of the tables to present and report the results in the following section (step 5).

## Results

### Description of studies

**Fig. 2:**
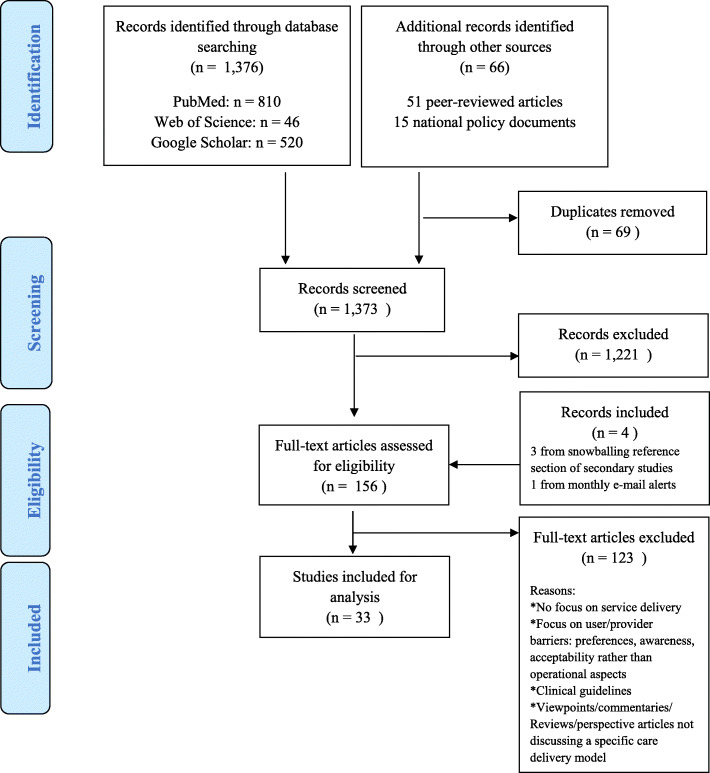
PRISMA flowchart for article selection

Our search and selection resulted in 156 records retained for full-text reading (Fig. 2). Of these, 86 articles were the result of tiab-screening from the peer-reviewed literature in three databases. Additionally, we identified 66 articles and/or abstracts through snowballing on references from primary resources, searching conference archives and government and institutional websites. Finally, we retrieved three records by snowballing references of secondary studies and one record via the monthly set e-mail alerts on the databases that were searched. After full-text reading, we selected and analysed 33 articles discussing PrEP service delivery models by the four required key components.

The majority of the articles comprised descriptive reports (*n* = 12) and pilot studies (*n* = 8). The included records were all published between 2014 and 2019, with the majority of all literature published between 2018 and 2019 (*n* = 25). The geographic focus of the analysed literature was centered in the USA (*n* = 16), where literature on the topic under study also first emerged. The majority of the literature originated from Northern America (*n* = 21). Eight articles had a focus on African countries, namely Kenya, Zimbabwe and South Africa. The remainder of the records reported on Western Europe (*n* = 5), Southeast Asia (*n* = 2), the Western Pacific (*n* = 2) and Latin America (*n* = 1).

See Table 2 for a complete overview of the included literature. A more detailed overview of each record’s study design and main findings is available in Additional file 5. A chronological overview of the literature is presented in Additional file 6.

**Table 2:** Overview of the included literature

#	Author & Year	Title	Study Type	Geographic Focus or Setting	PrEP Service Delivery Model		
					Service Delivery Setting	PrEP Provider	Delivery Channel
1	Finkenflügel et al., 2019 [22]	A Mobile Application to Collect Daily Data on Preexposure Prophylaxis Adherence and Sexual Behavior Among Men Who Have Sex With Men: Use Over Time and Comparability With Conventional Data Collection	Quantitative Usability & Feasibility Pilot Implementation Study	Amsterdam, The Netherlands	Research conducted in one STI outpatient clinic as part of the AmPrEP research study setting.	Study staff (health care worker training not specified)	3-monthly PrEP prescriptions obtained through in-person visits. In-between follow-up on adherence and sexual behavior through the use of a mobile application.
2	Fuchs et al., 2018 [23]	A Mobile Health Strategy to Support Adherence to Antiretroviral Preexposure Prophylaxis	Mixed Methods Usability, Acceptability & Feasibility Pilot Implementation Study	San Francisco & Chicago, USA	Research conducted in a research study setting as part of the iPrEx OLE study.	Study staff (health care worker training not specified)	3-monthly PrEP prescriptions obtained through in-person visits. In-between adherence support provided through the use of bidirectional text or e-mail messages.
3	Brown et al., 2018 [24]	Challenges and solutions implementing an SMS text message-based survey CASI and adherence reminders in an international biomedical HIV PrEP study (MTN 017)	Mixed Methods Pilot Implementation Study	USA, Thailand, Peru, South Africa	Research conducted in a research study setting as part of the MTN 017 phase 2 trial.	Study staff (health care worker training not specified)	Oral or rectal PrEP provided through in-person visits. SMS-CASI system used to provide real-time in-between visits adherence support
4	Puppo et al., 2019 [25]	Community-Based Care in the ANRS-IPERGAY Trial: The Challenges of Combination Prevention	Qualitative Adherence Evaluation Study	France & Canada	Research conducted in a research study setting as part of the ANRS-IPERGAY (OLE) trial.	Study staff (health care worker training not specified) prescribed oral PrEP. CBHW provide permanently available peer-based care.	PrEP prescriptions provided through in-person visits. Community-based support was permanently available as in-person visits with a personally assigned CBHW.
5	Sharma et al., 2018 [26]	Decentralizing the delivery of HIV pre-exposure prophylaxis (PrEP) through family physicians and sexual health clinic nurses: a dissemination and implementation study protocol	Protocol for a Pilot Implementation Study	Toronto, Canada	PrEP to be provided at family physician’s private practice or in sexual health clinics.	Family physicians and nurse practitioners in sexual health clinics provide PrEP prescriptions and related care	PrEP prescriptions provided through in-person visits with family physicians after receiving PICME or at the sexual health clinic for people with no family physician.
6	Siegler et al., 2019 [27]	Developing and Assessing the Feasibility of a Home-based Preexposure Prophylaxis Monitoring and Support Program	Implementation Pilot Study	San Francisco & Boston, USA	PrEP users receive care at home, replacing 3 out of 4 usually in-person visits	Clinicians (training not specified) at a variety of clinical settings provide PrEP prescriptions	STI/HIV sampling and testing kits sent to the user’s home with counseling and communication of test results over the phone. PrEP prescriptions sent to user’s home.
7	Liu et al., 2014 [28]	Early experiences implementing pre-exposure prophylaxis (PrEP) for HIV prevention in San Francisco	Descriptive Report	San Francisco, USA	1) PrEP demonstration project in a municipal STD clinic 2) PrEP program in a private health maintenance organization 3) PrEP delivery in a HIV-specific reproductive health program	1) Study staff (health care worker training not specified) 2) Prescription by HIV specialists, referrals by PCP 3) Health care worker training not specified	1) Oral PrEP provided through in-person visits in a study setting offering a range of sexual health services 2) Oral PrEP provided through regular in-person visits 3) Oral PrEP provided to HIV-negative women who are having sex with an HIV-positive male partner through in-person visits
8	Vuylsteke et al., 2018 [29]	High uptake of pre-exposure prophylaxis (PrEP) during early roll-out in Belgium: results from surveillance reports	Descriptive Report	Belgium	Oral PrEP provided through specialized HIV clinics.	Oral PrEP prescribed by HIV-specialists affiliated to specialized HIV clinics.	Oral PrEP provided through in-person visits via 11 centralized HIV reference centers across Belgium.
9	Girometti et al., 2018 [30]	Evolution of a pre-exposure prophylaxis (PrEP) service in a community-located sexual health clinic: concise report of the PrEPxpress	Descriptive Report	London, United Kingdom	Oral PrEP provided through a specialized sexual health clinic as part of the NHS-led 3-year IMPACT implementation trial.	Nurses provide oral PrEP per medical directive. Other trained healthcare workers are involved in monitoring and health promotion activities. Referral of complex cases to a physician-led consultation.	Oral PrEP provided through in-person visits. Clients can self-sample for STIs with test results provided via text message.
10	National AIDS & STI Control Programme (NASCOP), 2017 [31]	Framework for the Implementation of Pre-Exposure Prophylaxis of HIV In Kenya	National Implementation Framework	Kenya	Efforts to integrate oral PrEP service delivery horizontally in the Kenyan health system by strengthening existing services and reinforce linkage between complementary services such as reproductive health.	PrEP prescription and dispension by trained healthcare providers including physicians, clinical officers, nurses, pharmacists and pharmaceutical technologists.	Oral PrEP provided through in-person visits using both community-based and facility-based delivery models.
11	Hood et al., 2018 [32]	Getting pre-exposure prophylaxis to high-risk transgender women: lessons from Detroit, USA	Descriptive Report	Detroit, USA	Oral PrEP provided through a non-profit community health center delivering integrated and trans-friendly health services free of cost.	On-site local pharmacy provides PrEP regimens, in addition to hormonal replacement therapy (HRT). Training of other health care workers involved not specified.	Oral PrEP provided through in-person visits in a specialized clinical infrastructure responsive to the needs of transgender women.
12	Eakle et al., 2017 [33]	HIV pre-exposure prophylaxis and early antiretroviral treatment among female sex workers in South Africa: Results from a prospective observational demonstration project	Quantitative Implementation Study	Johannesburg & Pretoria, South Africa	Oral PrEP provided in a research setting at 2 urban clinics catering a population of sex workers.	PrEP prescriptions provided by a healthcare worker (training not specified). Sex Worker Program is run by nurses, community health workers and peer educators.	Oral PrEP provided through in-person visits integrated in a public health clinical service program providing primary healthcare to a population of sex workers.
13	Stekler et al., 2018 [34]	HIV Pre-exposure Prophylaxis Prescribing Through Telehealth	Descriptive Report	Seattle, USA	Oral PrEP provided at a community-based gay-friendly urban health center, targeting at risk persons without insurance.	PrEP prescriptions provided by physicians. Other care provided by trained HIV counselors.	Oral PrEP provided through in-person visits, either after consulting with a physician that is physically present, or after videoconference call with a physician.
14	Ortblad et al., 2019 [35]	HIV-1 self-testing to improve the efficiency of pre-exposure prophylaxis delivery: a randomized trial in Kenya	Protocol for a randomized non-inferiority trial	Thika, Kenya	Oral PrEP is provided in a research setting at an urban HIV clinic providing comprehensive HIV prevention and STI services.	PrEP prescriptions provided by study clinicians and PrEP is being dispensed by on-site pharmacists.	Oral PrEP provided through in-person visits. Introducing and testing of a service delivery model whereby HIV self-testing through oral vs. blood self-collection at home replaces one out of two of the quarterly clinic visits.
15	Tung et al., 2018 [36]	Implementation of a community pharmacy-based pre-exposure prophylaxis service a novel model for pre-exposure prophylaxis care	Descriptive Report	Seattle, USA	Oral PrEP provided through a community-based pharmacy.	Oral PrEP prescribed and provided by clinical pharmacist as per collaborative drug therapy agreement (CDTA) between physician and pharmacist. Referral to physician for complex cases.	Oral PrEP provided through in-person pharmacy visits under supervision of a physician medical director. STI samples collected through self-sampling, blood samples drawn by pharmacist.
16	Health Protection Scotland and Information Services Division, 2019 [37]	Implementation of HIV PrEP in Scotland: First Year Report	National Implementation Report	Scotland, United Kingdom	Oral PrEP provided exclusively in an existing network of specialized sexual health services.	Oral PrEP prescribed and dispensed by health care workers (training not specified)	Oral PrEP provided through in-person visits.
17	Ministry Of Health and Child Care, 2017 [38]	Implementation Plan for HIV Pre-Exposure Prophylaxis in Zimbabwe	National Implementation Plan	Zimbabwe	Plan to provide oral PrEP through phased facility roll-out with initial focus on existing facilities already serving key populations and other individuals at substantial risk of HIV acquisition.	Oral PrEP prescribed by health care workers in existing facilities providing HIV prevention services (provider training not specified).	Oral PrEP provided through in-person visits with focus on decentralizing PrEP services with a priority for high-incidence districts through existing systems.
18	Saxton et al., 2018 [39]	Implementing HIV pre-exposure prophylaxis (PrEP): Let’s not get caught with our pants down	Viewpoint	New Zealand	Oral PrEP provided through licensed HIV prescribers.	Oral PrEP prescribed and reimbursed through approved HIV prescribers. 3-monthly repeats can be provided by physicians and nurse practitioners after having received a PrEP training.	Oral PrEP provided through in-person visits.
19	Roesch et al., 2019 [40]	Implementing Pre-exposure Prophylaxis for HIV Prevention at an Urban Youth Clinic	Descriptive Report	Minnesota, USA	Oral PrEP provided at an urban community serving youth (age 11–24 years) regardless of ability to pay.	All PrEP care provided through nurse practitioners after having received a PrEP training course.	Oral PrEP provided through in-person visits
20	Pintye et al., 2018 [41]	Integration of PrEP Services Into Routine Antenatal and Postnatal Care: Experiences From an Implementation Program in Western Kenya	Pilot Implementation Study	Kisumu, Kenya	Oral PrEP provided in a research setting through a public or private sector antenatal or postnatal care clinic.	All PrEP-specific services provided by nurses within the ANC/PNC clinic. Option between co-delivery of PrEP service by same nurse offering maternal services or sequentially by a specialized PrEP nurse.	Oral PrEP provided through in-person visits.
21	Hoth et al., 2019 [42]	Iowa TelePrEP: A Public-Health-Partnered Telehealth Model for Human Immunodeficiency Virus Preexposure Prophylaxis Delivery in a Rural State.	Descriptive Report	Iowa, USA	PrEP care provided through videoconference in community settings or at people’s home.	TelePrEP pharmacists clinically assessed, educated and prescribed PrEP through videoconference. Clients were self-referred or referred by TelePrEP navigators when expressed interest in PrEP.	Oral PrEP was provided without need for in-person visits. All PrEP care was provided via videoconference, with laboratory testing performed in community laboratories and PrEP regimens sent to the user’s home.
22	Schmidt et al., 2018 [43]	Nurse-led pre-exposure prophylaxis: a non-traditional model to provide HIV prevention in a resource-constrained, pragmatic clinical trial	Descriptive Report	Sydney, Australia	Oral PrEP provided in a research setting at public clinics.	Trained and authorized registered nurses provide PrEP care and supply PrEP under standing order or following prescription by a physician.	Oral PrEP provided through in-person visits at public clinics participating in the EPIC-NSW trial.
23	Masyuko et al., 2018 [44]	Pre-exposure prophylaxis rollout in a national public sector program: the Kenyan case study	Case Study	Kenya	Oral PrEP provided in an integrated fashion in a variety of existing infrastructures such as HIV testing sites, HIV clinics, out-patient departments and maternal health care sites.	PrEP care can be initiated by a qualified and skilled health care provider with different (clinical) backgrounds.	Oral PrEP is provided through in-person visits.
24	Refugio et al., 2019 [45]	PrEPTECH: A Telehealth-Based Initiation Program for HIV Pre-exposure Prophylaxis in Young Men of Color Who Have Sex With Men. A Pilot Study of Feasibility	Quantitative Pilot Feasibility Study	San Francisco, USA	Oral PrEP provided through home-based telehealth visits.	PrEP care provided through an infectious disease physician over telephone. Clients receive STI sampling kits at home.	Oral PrEP is provided without the need for an in-person visit. Counseling and support is provided via telehealth visits, STI and HIV sampling is done via self-sampling and/or at nearby laboratories. PrEP regimens are sent to clients’ home.
25	Phanuphak et al., 2018 [46]	Princess PrEP program: the first key population-led model to deliver pre-exposure prophylaxis to key populations by key populations in Thailand	Descriptive Report	Thailand	Oral PrEP provided through 8 gay-friendly community health centers.	PrEP services, including HIV and STI testing, counseling and dispensing of regimens are being provided by trained key-population community health workers.	Oral PrEP provided through in-person visits in a community setting. Laboratory results are reviewed by a physician, complex cases and contra-indications for PrEP are referred to a physician. Visit reminders were sent via social networking platforms.
26	Bien et al., 2017 [47]	Reaching Key Populations: PrEP Uptake in an Urban Health Care System in the Bronx, New York	Descriptive Report	The Bronx, NYC, USA	Oral PrEP provided through a variety of clinical settings (predominantly primary care providers, next to sexual health clinics and women’s health centers), comprising together the urban health system of The Bronx.	PrEP care was provided through a variety of health care workers (training not specified).	Oral PrEP was provided through in-person visits.
27	Kamis et al., 2019 [48]	Same-Day HIV Pre-Exposure Prophylaxis (PrEP) Initiation During Drop-in Sexually Transmitted Diseases Clinic Appointments Is a Highly Acceptable, Feasible, and Safe Model that Engages Individuals at Risk for HIV into PrEP Care	Quantitative Feasibility and Acceptability Pilot Implementation Study	Denver, USA	Oral PrEP provided through a large metropolitan STD clinic.	PrEP care provided by a nurse practitioner or nurse together with a physician. A patient navigator provided PrEP education an PrEP was dispensed through an on-site pharmacy.	Oral PrEP was provided through in-person visits on the same day PrEP eligibility was first assessed.
28	Marcus et al., 2016 [49]	Successful Implementation of HIV Preexposure Prophylaxis: Lessons Learned From Three Clinical Settings	Review	San Francisco, USA	Oral PrEP provided in 3 different clinical settings in a metropolitan city.	Oral PrEP was provided by 1) infectious disease specialists or trained pharmacists 2) nurses in an STI clinic as part of a demonstration project 3) primary care practitioners, sending PrEP prescriptions to the preferred pharmacy of the client.	Oral PrEP was provided through in-person visits.
29	Walmsley et al., 2019 [50]	The PrEP You Want: A Web-Based Survey of Online Cross-Border Shopping for HIV Prophylaxis Medications	Quantitative Survey Study	Ontario, Canada	Oral PrEP provided through online ordering and shipping to a US mailbox.	Prescription for oral PrEP is obtained from a local Canadian health care provider and then shipped across the border to a US mailbox for pick-up.	Oral PrEP was provided without need for in-person visits. After obtaining a valid prescription, PrEP was ordered online and accessed using a border-crossing approach to bypass required co-payments in some Canadian jurisdictions.
30	Eccles-Radtke et al., 2015 [51]	Turning the Tide against AIDS by Preventing New HIV Infections: Initial Experience with Minnesota’s First PrEP Clinic	Descriptive Report	Minnesota, USA	Oral PrEP was provided at a local county medical center.	Oral PrEP was provided and dispensed on-site. Training of health care providers involved was not specified.	Oral PrEP was provided through in-person visits.
31	Liu et al., 2019 [52]	Randomized Controlled Trial of a Mobile Health Intervention to Promote Retention and Adherence to Preexposure Prophylaxis Among Young People at Risk for Human Immunodeficiency Virus: The EPIC Study	RCT efficacy study	Chicago, USA	Oral PrEP was provided in a research setting at a large public health clinic focused on HIV prevention.	Oral PrEP was delivered by on-site study clinicians (training not specified).	Oral PrEP was provided through in-person visits. Study participants of the intervention arm received PrEPmate, an mHealth application delivering information on PrEP, peer testimonials, online support forum, pill reminders and check-in messages.
32	National Department of Health, 2016 [53]	Guidelines for Expanding Combination Prevention and Treatment Options: Oral Pre-Exposure Prophylaxis (PrEP) and Test and Treat (T&T)	National (implementation) guidelines	South Africa	Oral PrEP is currently provided through existing sex worker programmes and linkage to primary health care facilities. Additional delivery mechanisms and target populations will be incorporated in a phased approach.	All healthcare providers affiliated with PrEP and ART service delivery should complete a PrEP implementation training program.	Oral PrEP is provided through in-person visits using current platforms of ART delivery.
33	O’Byrne et al., 2019 [54]	PrEP-RN: Clinical Considerations and Protocols for Nurse-Led PrEP	Review	Canada, USA	Oral PrEP is provided through a STI clinic.	Oral PrEP care is being provided by registered nurses as per medical directive. After 6–12 months of providing services at the STI clinic, patients are transferred to primary care for follow-up.	Oral PrEP is provided through in-person visits.

### Target population of PrEP services

#### Generic models

Seventeen articles focused on people at high risk of HIV acquisition without targeting a specific group [28–31, 36–39, 42–44, 47–49, 51, 53, 54]. Yet the description of people at risk of HIV in these records was often based upon differing criteria of eligibility. For instance, some records from Western countries (e.g. Canada, Scotland and Belgium) had additional behavioral criteria specified for MSM [29, 37, 54], which were largely absent in records from sub-Saharan Africa [31, 38, 44, 53]. Despite their focus on people at high risk of HIV, eight reports mentioned uptake of PrEP was mainly among MSM [28, 31, 36, 39, 44, 47–49].

#### Key population-specific models

**Fig. 3:**
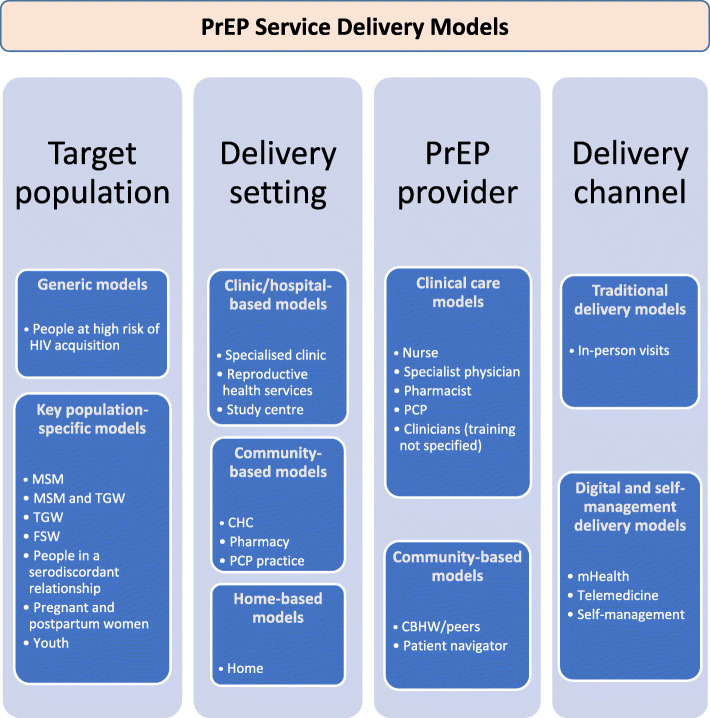
Description of different models for PrEP service delivery identified in the literature, according to the four key components: target population, delivery setting, PrEP provider, and delivery channel

**Fig. 4:**
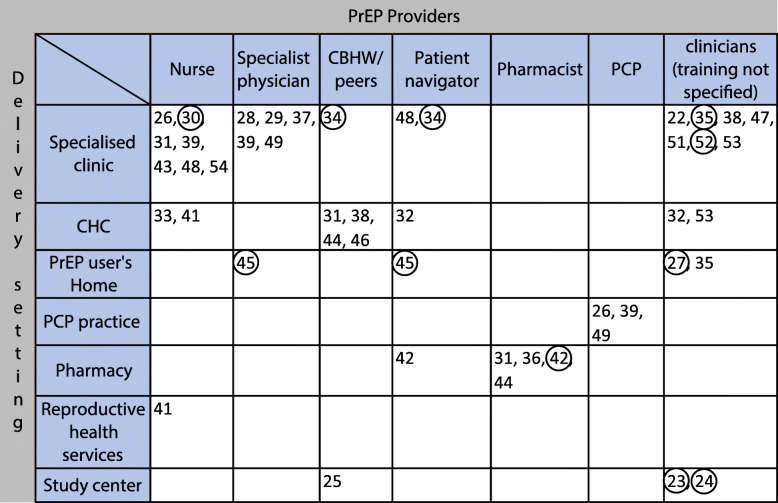
Position of the identified records according to delivery setting (vertical axis) and providers involved in PrEP care (horizontal axis). Encircled records reported an mHealth or telemedicine aspect to their used delivery channel for PrEP

**Fig. 5:**
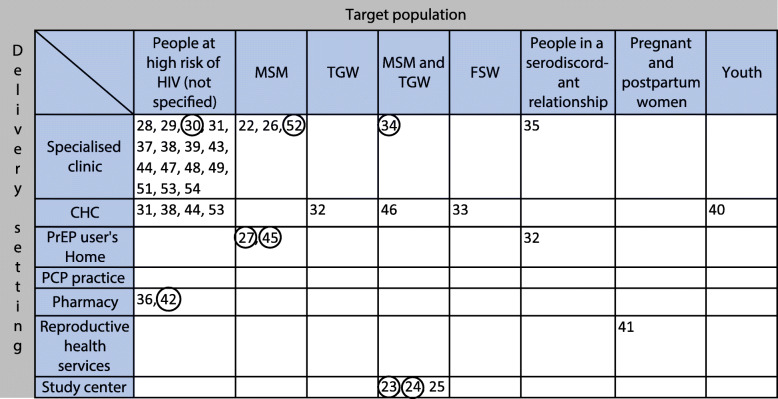
Position of the identified records according to delivery setting (vertical axis) and target population (horizontal axis) for PrEP care. Encircled records reported an mHealth or telemedicine aspect to their used delivery channel for PrEP

Six articles had an exclusive focus on MSM as a target group [22, 26, 27, 45, 50, 52]. Five articles had a focus on the combination of MSM and TGW [23–25, 34, 46]. One study focused exclusively on TGW [32], one on female sex workers (FSW) [33], one on men and women in a serodiscordant relationship [35], one on pregnant or postpartum women [41] and one study focused exclusively on youth (11–24 years old) at risk of HIV acquisition [40]. See Fig. 3 for an overview of the different PrEP service delivery model components in the included literature. See Figs. 4-5 for an overview of the overlap between key components of the PrEP service delivery models reported in the included records.

### Delivery settings: where is PrEP care being provided?

#### Clinic/hospital-based models

The majority of all articles (*n* = 19) mentioned PrEP services were available in specialised STI, HIV and/or sexual health clinics. The staffing of these clinics differed, yet was reportedly often nurse-led [26, 30, 31, 39, 43, 48, 54]. In high-resource settings, PrEP was more likely to be delivered in such specialised clinics, in particular in USA, Belgium, Scotland, England, Australia, New Zealand and Canada [26, 28–30, 37, 39, 43, 48, 54]. In reports from Kenya, Zimbabwe and South Africa, specialised clinic were mentioned as part of the existing facilities used to scale-up the provision of PrEP.

South Africa, specialised clinics were mentioned as part of the existing facilities used to scale-up the provision of PrEP [31, 35, 38, 44, 53]. The policy reports from the latter three African countries were the only identified records offering a nation-wide implementation plan for PrEP [31, 38, 53]. They discussed the integration of PrEP services in multiple strategically chosen (clinical) locations to increase access. Existing HIV clinics were but one possible delivery site for PrEP.

In one article, PrEP services were provided in a hospital setting by integrating PrEP in reproductive health services provided to pregnant and postpartum women in Kenya [41]. Lastly, sometimes PrEP was delivered as part of a study setting, with the clinical site of PrEP provision being referred to as ‘study centres’ [22–25].

#### Community-based models

Eight articles reported on people accessing PrEP services through a community health center (CHC) providing care adapted to the needs and demands of the community. These often included services for sexual health in general, including PrEP, and targeted TGW, MSM, FSW and youth in particular [32, 33, 40, 46]. In policy reports of Kenya, Zimbabwe and South Africa, CHCs were mentioned as a possible PrEP delivery site fitting their decentralisation strategy for the provision of PrEP [31, 38, 44, 53]. The CHCs in these settings focused on identifying people at high risk of HIV living in country regions with a high rate of new HIV diagnoses.

Two articles from the USA mentioned how community pharmacies had a role beyond the mere supply of PrEP by providing services such as assessing eligibility, and testing for HIV and STIs [36, 42]. In these two models, the existence of a legal framework made it possible for clinical pharmacists to deliver PrEP under remote clinician oversight. Also primary care practitioners (PCPs) were reported to deliver PrEP in their respective practices [49, 51].

#### Home-based models

Three articles described how people could receive home-based PrEP services, by performing parts of PrEP-related care themselves. This could include sampling for STIs and/or HIV testing [27, 35]. Clients and providers could engage in counseling services over the phone or through videoconference [45]. These examples highlighted how home-based care can be a way to deliver PrEP services by overcoming barriers towards access, such as anticipated (racial) discrimination by health care providers, distance and cost [27, 45].

### Provider options: who does what in PrEP care?

#### Clinical care models

The exact training of health care providers delivering PrEP services was not always mentioned in the included literature. These providers were however referred to as (study) clinicians, revealing their clinical background. Six articles mentioned all PrEP clients had been in contact with a specialist physician (either a specialist in infectious diseases, sexual health and/or HIV) to obtain their PrEP regimen [28, 29, 37, 39, 45, 49].

In nearly one-third of all articles (*n* = 10) PrEP services could be classified as ‘nurse-led’. In these models nurses were pivotal providers of PrEP-related services, with physicians consulted for ‘complex cases’ [26, 30, 31, 33, 39–41, 43, 48, 54]. The feasibility of a nurse-led approach was confirmed in different settings, from STI clinics to CHCs [26, 30, 39, 43, 48, 54]. The authors explained how such task-shifting strategy could be more cost-effective and allowed for decentralised care [40, 54]. Most of the tasks described in routine PrEP care, including counseling, eligibility screening, sampling for STIs and HIV, and providing adherence support, were performed by nurses and nurse practitioners. O’Byrne et al. reported on their experience with a nurse-led PrEP care model in a STI clinic in Canada, with a protocol for providing all PrEP-related services under medical directive (i.e. under supervision of a medical doctor) [54]. Kamis et al. outlined a model for nurse-based PrEP service delivery in a walk-in STI clinic, with a same-day PrEP initiation scheme [48].

Four articles mentioned pharmacists as key providers for PrEP services. They described how pharmacies in Kenya had a crucial role in identifying potential PrEP users in the community [31, 44]. Two other articles described how pharmacists in the USA delivered all PrEP-related services (eligibility screening, counseling, STI and HIV testing, PrEP dispensing and adherence support) under medical directive. In case of unusual lab results and/or PrEP-related complications, clients were referred to a physician [36, 42].

Also PCPs or family physicians were reported as the main provider of PrEP services. Primary care practitioners were able to prescribe subsidised PrEP and delivered related services in USA and Canada [26]. In New-Zealand PCPs could provide PrEP only after additional training and after consultation with a specialist physician [39].

#### Community-based models

Community-based health workers (CBHW) or peers were reportedly involved in PrEP service delivery. Their role varied, and involved mostly counseling and providing adherence support for PrEP users [25, 31, 38, 44]. One model in Thailand also described how peer lay providers screened for eligibility, sampled lab specimens, and provided PrEP regimens to users all at once [46]. The involvement of community workers or peers in PrEP delivery was therefore relatively more prevalent in records from LMICs.

In five articles, all from the USA, ‘patient navigators’ were mentioned as dedicated persons providing PrEP users with assistance in navigating insurance schemes and financial support structures [32, 34, 42, 45, 48].

### PrEP delivery channels: how to get PrEP to the people?

#### Traditional delivery models

Delivery channels were understood as platforms or media that were used to deliver PrEP services to the people. In most of the articles, PrEP care was provided through a ‘traditional’ face-to-face setting, meaning PrEP clients attended in-person to a service provider in a given physical setting [25, 26, 28–33, 35–41, 43, 44, 46–49, 51–54].

#### Digital and self-management models

Ten articles reported the use of ‘mHealth’ or ‘telemedicine’ as a way to engage with parts of the PrEP care. We defined mHealth as mobile applications allowing PrEP users to be actively engaged in their own health. Such applications were applied to increase adherence to PrEP regimens and/or to improve retention in care [22, 23, 52]. Other articles reported on communication between provider and PrEP client using mobile technology such as short message service (SMS), and telephone or videoconference calls (classified as ‘telemedicine’). Text messages (SMS) were used as a way to check on how PrEP clients were coping, as short adherence reminders, or to send the results of clients’ STI tests [30]. In the USA, videoconference or telephone calls between client and providers were used to perform aspects of PrEP care related to counseling, eligibility screening, medical history taking, adherence support and communication of test results [27, 34, 42, 45]. The use of models involving mHealth and telemedicine options for PrEP delivery was exclusively explored in high-resource settings, with the exception of one study on SMS support for PrEP users in Thailand, Peru and South Africa [24].

Self-management, including self-testing for HIV or self-sampling for STI testing, allowed PrEP clients to replace in-person clinic or laboratory visits by home-based self-care [27, 45]. A range of self-management options was found to be currently applied. Typically, at the one end, clients were attending regular 3-monthly in-person visits with in-between self-monitoring of adherence [22–24, 52]. At the other end, a completely home-based PrEP care model emerged, where PrEP users were not required to leave their house to continue on PrEP regimens [27, 45]. In between these two ends, models were described whereby one or more in-person visits were replaced by tele-visits or home-based self-care [34, 35, 42].

Finally, there was reporting of a model of informal PrEP delivery in Canada [50]. After obtaining a PrEP prescription by a local Canadian health care provider, PrEP was bought online and shipped to a mailbox in the USA. It was subsequently collected there in order to escape high medication costs in the Ontario jurisdiction in Canada, where PrEP is not (yet) subsidised by public funds.

## Discussion

This scoping review aimed to map and synthesise the literature on PrEP service delivery models worldwide. We systematically looked at four components of PrEP delivery models: the target population, the setting in which services were provided, the different provider options, and possible delivery channels through which PrEP was offered. We identified a range of possibilities within all of these components. The target population was found to range from specific sub-groups, mainly MSM, up to a less well defined population ‘at high risk of HIV acquisition’ or ‘youth’. Delivery settings differed in terms of the degree of centralisation of services, from highly centralised specialist clinics to offering PrEP at the individual’s home. Providers’ professionalisation varied from trained medical professionals to lay providers. Delivery channels ranged from traditional in-person visits to more innovative self-management options. How these components were combined and developed into a specific service delivery model in each of the identified records, was context-specific with large variations across different settings and populations.

This broad range of possible PrEP service delivery models was not only the result of care being adapted to users’ needs, as was also found in a recent scoping review by Hillis et al. [55]. This was also due to care being organised in an efficient way, using resources that were already available to address certain target groups. With more countries and settings implementing and adapting PrEP service delivery to fit their specific contexts, more options will certainly be developed. Ultimately, the most optimal delivery model of PrEP may therefore be one that best differentiates services according to the needs of different sub-groups of PrEP users while efficiently using available resources to target them. This notion of differentiated service delivery was defined by Grimsrud et al. as “*a client-centered approach that simplifies and adapts HIV services across the HIV care cascade to reflect the needs and preferences of various groups of people vulnerable to or living with HIV, while reducing unnecessary burdens on the health system*” [56]. Also WHO included a call for differentiated care in their most recent consolidated guidelines for ART delivery and for PrEP implementation alike [9]. The International AIDS Society (IAS) adopted a framework to guide decision-making towards differentiated ART delivery for key populations [57]. In this sense, we can already learn from service provision for people living with HIV (PLWH), where more experience exists with delivering differentiated care [58].

In many settings, PrEP delivery was integrated in existing services, such as the provision of antiretrovirals to PLWH, or sexual health care to populations at high risk of acquiring HIV [59, 60]. Especially in high-resource settings, PrEP services were commonly offered in a centralised and specialised way, namely through HIV, STI and sexual health clinics. This finding may reflect the reality that the first PrEP implementation studies were often organised within such specialised structures [61]. PrEP provision was subsequently scaled up in the same settings where initial experience with PrEP delivery was built. One of the lessons learned from the scale-up of centralised ART delivery models for PLWH, is that these settings may become overcrowded as demand grows [62]. Additionally, geographic accessibility issues and tailoring care to different sub-populations are other challenges identified in centralised clinical settings for ART delivery [62]. PrEP candidates, who usually are healthy, might even be less motivated than PLWH to travel long distances to health facilities or sit through long waiting periods to access care. However, we also found examples of de-centralised PrEP delivery, closer to the community. We identified some examples of community health centers catering for sexual and gender minority populations [32, 33, 40, 46]. These centers could serve as a valuable point to access PrEP care for these groups at high risk of HIV acquisition, since they may have gathered the necessary experience with delivering culturally-competent low-threshold care to such minority groups [63]. Also community pharmacies in the USA were mentioned as settings where PrEP delivery was considered feasible [36, 42]. However, it remains to be determined which particular groups or communities will be adequately reached by such community services, and whether providing long-term follow-up in such settings will prove to be successful.

In terms of PrEP providers, we found evidence for the implementation of task-shifting strategies, enabling non-physician profiles to deliver PrEP services. Particularly nurse-led PrEP delivery was reported to be feasible to organise [30, 33, 40, 43, 48, 54]. In this model, nurses and nurse practitioners provided PrEP care according to a protocol under medical directive. Especially in settings already delivering ART, task-shifting to nurse-led PrEP delivery could be considered as an efficient use of human resources and may even be cost-effective [54]. As PrEP was reported to be very safe and in general very well tolerated, shifting PrEP provision towards non-traditional health care settings and even lay providers becomes a reality. In the USA, clinical pharmacists operate within a legal framework allowing them to provide clinical care under specialised supervision. Therefore, they are in a good position to provide PrEP services for people who experience barriers to visit other medical providers [36, 42]. Also members of the community and of key populations can be involved in the delivery of PrEP or related services [25, 31, 38, 46]. Especially in contexts where community health workers have historically been more involved in the delivery of (HIV) services, such as sub-Saharan Africa and Thailand, task-shifting to lay providers might be more acceptable and successful for PrEP delivery alike [64, 65].

In the majority of the reports, PrEP was delivered through in-person visits by a provider in a given physical setting. Yet, we also identified the use of mobile applications (mHealth) and telemedicine as strategies to overcome issues of accessibility to regular health care services, as also mentioned by Hillis et al. [34, 42, 45, 55]. Moreover, these strategies were also applied to empower individuals’ active involvement in their own health status [22–24, 52]. Indeed, geographic disparities have been reported in access to PrEP care, and young MSM of color reported anticipated stigma and medical mistrust as additional barriers to access PrEP services [66, 67]. Limiting the number of in-person visits through home-based PrEP care using mHealth and telemedicine technology, provides in that sense a rationale to overcome some of the barriers described above. In addition, they could reduce the pressure on – sometimes overburdened – health care settings [27]. However, a challenge of these non-facility-based PrEP delivery models remains the need for continued regular HIV testing to confirm PrEP eligibility over time. Self-tests for HIV may offer a way forward to complement home-based PrEP care. Yet issues remain with cost and reliability of test results, especially during the window period of an acute HIV infection [68, 69]. Moreover, it should be clearly safeguarded that the introduction of mHealth and other self-management interventions does not increase inequity in access to PrEP care. This is especially a concern for those who are less literate or lack a degree of self-efficacy to use such applications, or experience other barriers towards its uptake.

### Recommendations for future research

More research is needed into different needs and preferences of sub-groups of PrEP users (e.g. ethnic and other minority groups) to adapt care accordingly. Indeed, it is unlikely that all PrEP users engage with PrEP in the same way. Therefore some do not require the same package of services nor the same level of intensity of health facility visits and follow-up than others [9].

Multiple forms of differentiated PrEP services are currently implemented, but it is unclear to what extent they are adapted to the individual characteristics of PrEP users.

We identified only few studies that went beyond more ‘traditional’ PrEP provider options and included other non-specialist profiles and non-health care workers in the scope of PrEP care. Further research should therefore continue to focus on how to best engage, motivate and educate other health care workers, such as primary care physicians and lay providers. Insights in how to increase cooperation between different providers to integrate PrEP provision successfully into their practice, could present a way forward [18].

In addition, there was a paucity of literature on integrated services, going beyond stand-alone PrEP delivery interventions. Yet PrEP services should ultimately be effectively integrated into services able to respond to potential syndemics in PrEP users such as sexualised drug use and mental health problems [70, 71]. Additionally, there is reportedly a high unmet HIV prevention need among sub-Saharan African women attending family planning services [72]. So in order to really fulfill the true potential of PrEP, there is a need to discuss and implement strategies focused on integrating PrEP within wider sexual and reproductive health care and prevention services. Studies describing the national PrEP implementation programs of Kenya, Zimbabwe, and South Africa did report on such integrated services and how they could be scaled-up [31, 38, 53]. Yet, whether they respond to the needs of different key populations still remains to be evaluated.

### Limitations

We acknowledge that there are limitations to the degree of comprehensiveness of this review. Due to a lag in indexing more recent articles to the literature databases that were searched, we might not have been able to capture fully the latest evolutions on this topic. Much of the experience with service delivery of PrEP in a real-world setting might also not been reported yet in the peer-reviewed literature, or elsewhere. We recognize that PrEP is a relatively new intervention and experience with its delivery is still growing as we write.

Secondly, we narrowed the scope of this review to a certain understanding of ‘PrEP service delivery models’ that made a literature search operational and feasible. Hereby, we missed articles discussing other important (health system) aspects of PrEP service delivery such as cost (e.g. insurance schemes and reimbursement policies), the legal and policy structures in which PrEP delivery operates, and the specific content and data and monitoring systems of PrEP care programs. Yet, some of these topics were recently reviewed elsewhere [55].

## Conclusions

We conducted a scoping review on PrEP service delivery models, resulting into an inventory of existing models and a synthesis of what is known about the target population, the settings in which PrEP is being delivered, PrEP providers’ profiles and the respective delivery channels. These settings were mainly found to be centralised in clinical settings, yet examples of highly de-centralised community-based and home-based PrEP care exist. PrEP providers were mainly clinical health care workers, with community health workers and lay providers identified only in a minority of the literature. Delivery channels for PrEP ranged from traditional in-person visits to more innovative self-management options. Future research should focus on how integrated services could provide differentiated PrEP care accounting for users’ individual needs within a combination prevention approach.

## Supplementary information

**Additional file 1.** Detailed description of the search strategy. Outline of how the different academic databases were searched in a systematic manner.

**Additional file 2.** Inclusion criteria for article selection. Overview of the inclusion criteria for article selection during tiab-screening and full-text reading.

**Additional file 3.** Measurement and interpretation of the Kappa coefficient. Description of how the Kappa coefficient to assess inter-rater variability of the selection decisions of the two reviewers was measured and interpreted.

**Additional file 4.** Data extraction sheet. Outline of all study characteristics that were systematically documented for each included record.

**Additional file 5.** Detailed overview of study design and main findings of the included literature. More detailed insight into the study design of the included records and their main findings.

**Additional file 6.** Overview of the evolution of the included literature. Overview of year of publication, geographical focus, and publication type for all included records.

**Additional file 7.** PRISMA extension for Scoping Reviews (PRISMA-ScR) checklist. Completed checklist applied to the manuscript.

## Data Availability

All data generated or analysed during this study are included in this published article, or in primary research articles to which references were made. Additional data are available in the supplementary files listed below:

## Abbreviations

AGYW: Adolescent girls and young women
ART: Antiretroviral therapy
CBHW: Community-based health worker
CHC: Community health center
FSW: Female sex worker
HIC: High-income country
HIV: Human immunodeficiency virus
IAS: International AIDS society
LMIC: Low-and-middle income country
MSM: Men who have sex with men
PCP: Primary care practitioner
PLWH: People living with HIV
PrEP: Pre-exposure prophylaxis
SMS: Short message service
STI: Sexually transmitted infection
TGW: Transgender woman
WHO: World health organization
