# Supplementary material for: The current landscape of pre-exposure prophylaxis service delivery models for HIV prevention: a scoping review
Source: BMC Health Serv Res. 2020 Jul 31;20:704. doi: 10.1186/s12913-020-05568-w (PMC7395423; doi:10.1186/s12913-020-05568-w)
Supplement: Supplementary file 1 — Additional file 1. Detailed description of the search strategy. Outline of how the different academic databases were searched in a systematic manner. [file 12913_2020_5568_MOESM1_ESM.pdf]

1. **Detailed search strategy.**

	Keyword	Search String	Search Outcome
**#1**	HIV	HIV[MeSH] OR HIV[tiab]	311,083 hits
**#2**	PrEP	"Pre-Exposure Prophylaxis"[MeSH] OR "pre-exposure prophylaxis"[tiab] OR "preexposure prophylaxis"[tiab] OR "prep"[tiab]	5,759 hits
**#3**	Health services delivery	"Delivery of Health Care"[MeSH] OR "Drug Delivery Systems"[MeSH] OR "health service provision"[tiab]	1,156,295 hits
	#1 AND #2 AND #3		828 hits
		Sorted 'Best Match'	829 hits
		+ Filter 'Humans'	818 hits
		+ Filter 'English'	810 hits

Search String adapted to different databases:

Database	Search String	Additional Filters	Outcome
MEDLINE (PubMed)	(HIV[MeSH] OR HIV[tiab]) **AND** ("Pre-Exposure Prophylaxis"[MeSH] OR "pre-exposure prophylaxis"[tiab] OR "preexposure prophylaxis"[tiab] OR "prep"[tiab]) **AND** ("Delivery of Health Care"[MeSH] OR "Drug Delivery Systems"[MeSH] OR "health service provision"[tiab])	Human<br>English	810 hits
WoS	TS=("hiv" OR "human immunodeficiency virus") **AND** TS=("prep" OR "pre-	/	46 hits

	exposure prophylaxis” OR “preexposure prophylaxis”) **AND** TS=(“delivery of care” OR “delivery of services” OR “health services organi*ation” OR “health system*” OR “health*care delivery”)		
Google Scholar	("hiv" OR "human immunodeficiency virus") **AND** ("prep" OR "preexposure prophylaxis" OR "pre-exposure prophylaxis") **AND** ("healthcare delivery")	2012-2019	520 hits
Title and abstracts extracted to EndNote and monthly e-mail updates set.<br>15 duplicates (PubMed-WoS) were removed (856-15=841).<br>54 duplicates (Scholar-PubMed/WoS) were removed (1 361-54=1 307).			
