# Supplementary material for: The current landscape of pre-exposure prophylaxis service delivery models for HIV prevention: a scoping review
Source: BMC Health Serv Res. 2020 Jul 31;20:704. doi: 10.1186/s12913-020-05568-w (PMC7395423; doi:10.1186/s12913-020-05568-w)
Supplement: Supplementary file 2 — Additional file 2. Inclusion criteria for article selection. Overview of the inclusion criteria for article selection during tiab-screening and full-text reading. [file 12913_2020_5568_MOESM2_ESM.pdf]

2. Inclusion and exclusion criteria for study selection.

**Inclusion criteria**	Type of articles	- Full-text peer-reviewed research articles (primary and secondary research)<br>- Grey literature (e.g. reports and book chapters)
	Language	- English language
	Geographic context	- Worldwide
	Timing	- No restrictions
	PrEP delivery model	PrEP provision in real world setting with mentioning of:<br><br>- Infrastructural setting of PrEP delivery<br>AND<br>- Target population for PrEP<br>AND<br>- PrEP provider<br>AND<br>- Used delivery channel

**Exclusion criteria**	- Non-oral administration methods of PrEP<br>- Articles focusing on steps in the PrEP care continuum not related to PrEP provision (e.g. creating awareness and assessing eligibility)<br>- Non-research articles not discussing PrEP service delivery as its main focus.<br>- Demonstration projects not studying PrEP service delivery aspects as its main focus.
