# Supplementary material for: The current landscape of pre-exposure prophylaxis service delivery models for HIV prevention: a scoping review
Source: BMC Health Serv Res. 2020 Jul 31;20:704. doi: 10.1186/s12913-020-05568-w (PMC7395423; doi:10.1186/s12913-020-05568-w)
Supplement: Supplementary file 3 — Additional file 3. Measurement and interpretation of the Kappa coefficient. Description of how the Kappa coefficient to assess inter-rater variability of the selection decisions of the two reviewers was measured and interpreted. [file 12913_2020_5568_MOESM3_ESM.pdf]

3. **Measurement and interpretation of the Kappa coefficient.**

Table 1. Degree of Agreement Between Two Reviewers.

	**Reviewer 2 reject**	**Reviewer 2 accept**	**TOTAL**
**Reviewer 1 reject**	73	0	73
**Reviewer 1 accept**	8	8	16
	81	8	89

Table 2. Measurement of Kappa coefficient.

**(P0) Observed Agreement**	**(PE) Expected Agreement**	**K**
0,910	0,762	0,621

Table 3. Interpretation of Kappa coefficient.

**Interpretation**	
**Value of K**	**Strength of agreement**
< 0.20	Poor
0.21-0.40	Fair
0.41-0.60	Moderate
0.61-0.80	Good
0.81-1.00	Very good
