# Supplementary material for: The current landscape of pre-exposure prophylaxis service delivery models for HIV prevention: a scoping review
Source: BMC Health Serv Res. 2020 Jul 31;20:704. doi: 10.1186/s12913-020-05568-w (PMC7395423; doi:10.1186/s12913-020-05568-w)
Supplement: Supplementary file 4 — Additional file 4. Data extraction sheet. Outline of all study characteristics that were systematically documented for each included record. [file 12913_2020_5568_MOESM4_ESM.pdf]

4. Data extraction sheet.

Author
Date
Country, region and/or city where research was conducted
Research tradition (QUAL, QUAN, Mixed Methods)
Study type
Research purpose
Time frame in which research was conducted
Target population for PrEP
Health care setting in which PrEP was provided
PrEP provider characteristics
Delivery channels used for PrEP
Main findings
