# Supplementary material for: The current landscape of pre-exposure prophylaxis service delivery models for HIV prevention: a scoping review
Source: BMC Health Serv Res. 2020 Jul 31;20:704. doi: 10.1186/s12913-020-05568-w (PMC7395423; doi:10.1186/s12913-020-05568-w)
Supplement: Supplementary file 5 — Additional file 5. Detailed overview of study design and main findings of the included literature. More detailed insight into the study design of the included records and their main findings. [file 12913_2020_5568_MOESM5_ESM.pdf]

5. Detailed overview of study design and main findings of the included literature.

Author & Year	Study Design/Study Type	Subject Under Study		Time frame	Purpose of the article	Main Findings
		n (if reported)	target group			
Finkenflügel et al. , 2019	Quantitative usability and feasibility substudy in an open-label demonstration project (AmPrEP)	374	MSM on PrEP	12 months	To assess the feasibility and actual use of a mobile application by PrEP users in a demonstration project as a valid strategy to inform the provider about sexual behavior and adherence during PrEP intake.	The app was frequently used among PrEP users. App data regarding adherence and sexual behavior were concordant to 3-monthly questionnaire data. No information was provided on content of PrEP care provided during study visits.
Fuchs et al. , 2018	Pilot implementation study using mixed-methods, embedded in the iPrEx open-label extension (OLE) study	52	MSM & TGW on PrEP	12 weeks	To evaluate whether a mobile health intervention (iText) that utilized weekly bidirectional text or e-mail support messages to encourage preexposure prophylaxis (PrEP) adherence is effective in supporting adherence, acceptable for users and feasible to implement.	The intervention was reported as highly acceptable (particularly among young MSM and MSM of color) and demonstrated promising effects on PrEP adherence (77% reduction in missed doses on pill counts). Study staff deemed the intervention feasible to administer and thought it can be incorporated readily into clinic flow.
Brown et al. , 2018	Implementation study identifying challenges and solutions of an SMS-CASI system for adherence support in a phase 2 trial for microbicide PrEP use.	187	MSM & TGW on oral or rectal PrEP	27 weeks	To evaluate the safety and feasibility of incorporating a SMS-CASI system as real-time adherence support measure for oral and rectal PrEP and to identify operational challenges & provide concomittant solutions.	Implementation challenges included: high message costs; poor data access; slow data cleaning and analysis; difficulty reporting information to sites; a need for better participant privacy and data security; and mitigating variability in system performance across sites. Solutions to mitigate these challenges were offered.
Puppo et al. , 2019	Qualitative evaluation study embedded in the ANRS-IPERGAY (OLE) trial	83	MSM & TGW on PrEP	ANRS-IPERGAY: 16 months. ANRS-IPERGAY OLE: 1 year	To investigate the community-based support provided in the ANRS-IPERGAY trial and, more specifically, examine its relationship—in terms of integration, coexistence, and influence—with adherence to PrEP intake by HIV-negative MSM.	CBHW-participant relationship very likely had an important influence on adherence to PrEP, as many participants rated the peer support as one of the most important elements of PrEP adherence through stimulating empowerment, self-efficacy and self-management.

Author & Year	Study Design/Study Type	Subject Under Study		Time frame	Purpose of the article	Main Findings
		n (if reported)	target group			
Sharma et al. , 2018	Protocol for a mixed-methods pilot implementation study of 2 complementary strategies to decentralize PrEP delivery	Not pre-specified	gbMSM	18 months	To quantify the uptake of PrEP achieved using each of the two decentralization strategies and to assess the acceptability, feasibility and cost of each intervention.	Not yet available.
Siegler et al. , 2019	Pilot implementation study introducing home-based PrEP care visits, together with a behavioral survey	55	MSM on PrEP	Not specified	Assessing the feasibility and acceptability of a home-based PrEP care model replacing 3 of the 4 annual in-person follow-up visits.	The intervention was rated as acceptable and in demand for future use. Individual components of the intervention were highly rated, and all but 4 participants were able to collect sufficient amounts of specimen for required laboratory testing. More than one-third of participants reported that they would be more likely to remain on PrEP if PrEP@Home was available.
Liu et al. , 2014	Descriptive Report, underscored with quantitative data	3	Different parallel PrEP delivery platforms presented in the same city with a high incidence of HIV in MSM	Not specified	To describe early experiences with PrEP uptake and delivery in the first year of PrEP implementation post-FDA approval in three different clinical settings in San Francisco, California.	PrEP implementation in these 3 clinical settings was deemed feasible. Need to increase consumer knowledge on PrEP. Risk perception and safety concerns of potential candidates are to be addressed in order to increase uptake and adherence. Ensuring adequate clinic capacity and sustainable delivery of PrEP is critical to addressing ongoing high demand for PrEP. PrEP stigma to be addressed by both providers and communities to maximize impact.
Vuylsteke et al. , 2018	Descriptive Report on number of PrEP initiations in 7 Belgian HIV Reference Centers one year after national roll-out using quantitative data	7 HIV Reference Centers	All persons at high risk of HIV acquisition (no subgroups specified)	Up to February 2018	To determine the uptake of daily and event-driven PrEP in Belgium during the first 9 months of national PrEP roll-out.	The uptake of PrEP in Belgium since the start of the roll-out in June 2017 has been high (1050 initiations), and almost entirely limited to MSM, of whom 43% initially prefer a non-daily regimen.

Author & Year	Study Design/Study Type	Subject Under Study		Time frame	Purpose of the article	Main Findings
		n (if reported)	target group			
Girometti et al. , 2018	Descriptive Report on the scale-up of Dean Street 56 sexual health express clinic needed to include the 1700 assigned trial participants for the IMPACT trial	1700	All persons at high risk of HIV acquisition (no subgroups specified)	Ongoing	Report on the scale-up needed at Dean Street 56 to move from a doctor-led to a nurse-led model of PrEP delivery needed to manage the commissioned 1700 enrollments for the IMPACT trial.	PrEP advice and monitoring were easily accommodated in the 56 Dean Street sexual health services, but did require additional training of healthcare workers and approval for nurse prescribing and dispensing of the drug in order to achieve the target of 1700 enrollments, which still fell short of the demand for PrEP as additional PrEP candidates keep coming forward.
National AIDS & STI Control Programme (NASCOP), 2017	Framework to guide the PrEP implementation efforts in Kenya	Not applicable	All persons at high risk of HIV acquisition (no subgroups specified)	2017 and beyond	The objectives of the framework are in line with the provision of a holistic HIV prevention package in Kenya. This is through promoting acceptability of PrEP, ensuring availability and access to PrEP and related services and overall health systems strengthening.	Framework to organize PrEP care in Kenya, informed by the results of several demonstration projects in the country. PrEP will be delivered through both community-based and facility-based delivery models, using strengthening of existing infrastructure. Service delivery points initiating clients on PrEP must meet a minimum set of criteria outlined in the framework.
Hood et al. , 2018	Descriptive Report of a PrEP delivery model targeting TGW, underscored by data from a cross-sectional online survey	126	TGW	8 months	Survey aiming to describe the risk profile of Detroit TGW, the proportion of TGW with at least one PrEP indication and perceptions of and experiences with PrEP among TGW in a trans-friendly community-based health centre in Detroit.	Specialised clinical infrastructure that is responsive to the specific needs of TGW may be needed to expand PrEP. This TGW friendly delivery model in Detroit provides PrEP services in conjunction with HRT, integrating clinical and social services, with flexible scheduling and drop-in, on-site PrEP dispensing and the creation of a trusted environment.
Eakle et al. , 2017	Prospective observational cohort designed as a real-world implementation study	511	FSW	27 months	To investigate whether female sex workers would take up and use PrEP (as part of a combination prevention package) and early ART if these interventions were offered to them in existing sex worker specific clinics.	PrEP can be aligned with existing health service programming for FSWs safely, without significant behaviour change, and with high rates of uptake, but also high rates of lost-to-follow-up and with expected cost reduction in routine settings at scale.

Author & Year	Study Design/Study Type	Subject Under Study		Time frame	Purpose of the article	Main Findings
		n (if reported)	target group			
Stekler et al. , 2018	Descriptive report underscored with data from a cross-sectional quantitative study	48	MSM & TGW	9 months	To assess the feasibility and acceptability of a physician-led Telemedicine approach for PrEP prescribing leaving counseling and laboratory specimen collection to trained HIV counselors.	Feasibility and acceptability of a telehealth approach to PrEP prescribing that promotes HIV counselors as PrEP experts was shown. This model has several advantages, including the flexibility it provides to the specialist and the transition of time and effort to less-expensive staff who are content experts in HIV and sexual health.
Ortblad et al. , 2019	Protocol for a three-arm randomized non-inferiority trial with qualitative part (JiPime-JiPrEP)	495	HIV-1 uninfected men and women in a serodiscordant relationship and HIV-1 uninfected women not in a serodiscordant relationship	12 months	To assess the feasibility, acceptability, effectiveness and safety of HIVST as a model to support PrEP delivery in Kenya. The findings can inform policy aimed at improving the efficiency of PrEP implementation and scale-up in Kenya.	Not yet available.
Tung et al. , 2018	Descriptive report of a pharmacist-managed community PrEP clinic	695	All persons at high risk of HIV acquisition on PrEP (no subgroups specified)	36 months	Describing early experiences with a CDTA protocol allowing pharmacists to initiate and manage oral PrEP under the supervision of a physician medical director.	A pharmacist-managed PrEP clinic proved to be a successful alternative model of PrEP care, with high initiation rates and low drop-out and lost-to-follow-up rates. This may benefit individuals who do not access PrEP in traditional health care settings or where PrEP access is scarce.
Health Protection Scotland and Information Services Division , 2019	Report describing the implementation, monitoring, uptake, and preliminary outcomes associated with the first year of PrEP services roll-out in Scotland	Not applicable	All persons at high risk of HIV acquisition (no subgroups specified)	1 year	To document main outcomes of monitoring and evaluation one year after PrEP roll-out through NHS in Scotland.	Scotland is one of the first countries worldwide to have successfully established a PrEP service which is truly national, free at the point of delivery and, to date, generally accessible to most deemed at high risk of acquiring HIV. The success of the service implementation, and the cumulative nature of PrEP uptake, means that pressures on sexual health services will continue to increase unless additional resources or efficiencies are identified.

Author & Year	Study Design/Study Type	Subject Under Study		Time frame	Purpose of the article	Main Findings
		n (if reported)	target group			
Ministry Of Health and Child Care , 2017	National Implementation Plan for PrEP roll-out	Not applicable	All persons at high risk of HIV acquisition (no subgroups specified)	Not applicable	To come up with an implementation plan to provide oral PrEP as part of a combination HIV prevention approach, to people at high risk of HIV infection between 2018 and 2020 in order to contribute to the reduction of HIV incidence in Zimbabwe.	Focus on decentralization of PrEP services with a priority for high-incidence districts through existing systems already serving key populations using a HIV combination prevention approach. Involvement of both public and private sector to expand services. Objectives and targets set to meet this goal.
Saxton et al. , 2018	Viewpoint article in which the New Zealand PrEP delivery model is described	Not applicable	All persons at high risk of HIV acquisition (no subgroups specified)	Not applicable	To review the rationale for PrEP in New Zealand and to identify barriers to rapid implementation.	After fast-track funding for PrEP as a biomedical HIV prevention tool in New Zealand, the aim should be to implement it with the same pro-active spirit. Widening the range of PrEP prescribers at initial application, e.g. by including all sexual health physicians and registered medical doctors who have completed an accredited PrEP prescribing course, would better help reduce prescribing bottlenecks and improve access.
Roesch et al , 2019	Descriptive report of a quality improvement project using a PCDA-cycle approach	Not applicable	Youth (11-24 years old)	12 months	The primary aim of the project was to increase access to PrEP for high-risk youth and to prevent HIV infections in this population by educating care providers and implementing a PrEP protocol for nurse practitioners at an urban youth clinic.	Pre-exposureprophylaxis was successfully implemented at an urban Midwest youth clinic, increasing access for local youth. However, uptake among young people was low (n=10) and less than one-third of all PrEP initiators attended any follow-up visits.
Pintye et al. , 2018	Report on a PrEP implementation program (PrEP Implementation for Young Women and Adolescents, PrIYA) using clinical flow mapping in the PrIYA	Not applicable	HIV-negative pregnant or postpartum women	Not specified	To evaluate the work flow patterns and additional staff time associated with integrating PrEP into ANC/PNC services.	2 feasible clinic approaches for integrating PrEP delivery within routine ANC/PNC with moderate additional time required for initiating willing clients on PrEP were observed and described. Innovative approaches for increasing efficiencies and PrEP follow-up could further optimize PrEP delivery in ANC and PNC in high HIV burden settings.

Author & Year	Study Design/Study Type	Subject Under Study		Time frame	Purpose of the article	Main Findings
		n (if reported)	target group			
Hoth et al. , 2019	Descriptive report of a retrospective process evaluation using descriptive statistics	186	People reffered for PrEP from STI clinics/HIV testing services or self-referral through TelePrEP navigators	9 months	To describe an effective innovative strategy to improve PrEP delivery in rural and smaller urban areas where barriers include long distances to PrEP providers, limited availability of healthcare providers and high level of stigma surrounding HIV.	This report shows feasibility of a Tele-health approach, referring PrEP candidates by local public health departments for videoconference PrEP care by pharmacists and dispensing PrEP regimens sent at home. Satisfaction and acceptability of the services by the users were not assessed in this study.
Schmidt et al. , 2018	Descritptive report of a nurse-led model for PrEP in the EPIC-NSW trial in Australia	Not applicable	Eligible PrEP candidates in the EPIC-NSW trial	Not applicable	To describe the feasibility of a model for PrEP care, expanding the role of nurses in HIV care, including prescribing antiretroviral therapy (ART) for treatment and PrEP to overcome doctor shortages and other resource limitations.	A nurse-led model for PrEP delivery has been implemented successfully in public clinics in 10 of 14 local health districts, with widespread support and no serious safety events reported. Acceptability has not formally been assessed. With the increasing importance of PrEP as an HIV prevention tool, non-traditional models of care, including nurse-led PrEP, are needed.
Masyuko et al. , 2018	Case Study on wide-scale roll-out of PrEP in Kenya	Not applicable	All persons at high risk of HIV acquisition (no subgroups specified)	1 year after roll-out	To detail Kenya's experience of PrEP roll out as a national public sector program.	PrEP is now offered in over 900 facilities country-wide. There were over 14000 PrEP users 1 year after launching PrEP. Kenya is hereby the first African country to rollout PrEP as a national program in the public sector.
Refugio et al. , 2019	Longitudinal prospective cohort pilot study	25	HIV-negative YMSM of color	180 days	To pilot study the feasibility and acceptability of a telehealth approach to overcome barriers such as stigma, cost, adherence concerns and medical mistrust and increase access to PrEP for YMSM.	Telehealth programs such as PrEPTECH increase PrEP access for YMSM of color by eliminating barriers inherent in traditional clinic-based models.

Author & Year	Study Design/Study Type	Subject Under Study		Time frame	Purpose of the article	Main Findings
		n (if reported)	target group			
Phanuphak et al. , 2018	Descriptive report of a key-population led PrEP service delivery program in Thailand	1697	MSM and TGW	24 months	To report on the feasibility, acceptability and safety of a unique KP-led PrEP program serving MSM and transgender women (TGW) in Thailand.	A KP-led same-day PrEP initiation program successfully delivered PrEP to MSM and TGW. Innovative retention supports are needed, especially for TGW and those who are young or with lower education levels. To scale-up and sustain KP-led PrEP programs, strong endorsement from international and national guidelines is necessary.
Bien et al. , 2017	Descriptive report of a retrospective clinical database and chart review	108	All persons at high risk of HIV acquisition (no subgroups specified)	59 months	Little is known about PrEP initiation in large health care systems offering care across a spectrum of clinical settings. This study describes PrEP uptake within an integrated health care system in the Bronx, New York, an urban area with one of the largest HIV epidemics in the U.S.	A large increase in PrEP prescribing was observed over time in a HIV high-incidence urban health system, but overall PrEP prescribing was low. PrEP prescriptions occurred across a range of clinical settings, and we found that most PrEP prescribing occurred in primary care centers, women's health centers and a single sexual health center.
Kamis et al. , 2019	Longitudinal prospective quantitative feasibility and acceptability implementation study	100	All persons at high risk of HIV acquisition (no subgroups specified)	30 days	To determine whether a same-day approach to PrEP initiation could be feasible, acceptable and succesfully implemented in a busy, urban and public STD clinic.	Study suggests STD clinic-based, same-day PrEP initiation is acceptable, feasible, safe, and links a high proportion of individuals into ongoing PrEP care with a PrEP provider at participating clinics. Additional resources may be needed to support low-income individuals' retention in care as only low income was independently associated with attending a PrEP follow-up appointment.
Marcus et al. , 2016	Review of 3 PrEP delivery models in different clinical practices	Not applicable	Not specified	Not applicable	To describe 3 models of PrEP service delivery drawing on the experience of 3 different clinical settings in San Francisco (and 1 STI clinic in Miami) offering PrEP and discuss strengths and weaknesses of each model.	These early implementation experiences demonstrate that PrEP can be successfully delivered across a variety of settings and highlight strategies to streamline PrEP delivery in clinical practice.

Author & Year	Study Design/Study Type	Subject Under Study		Time frame	Purpose of the article	Main Findings
		n (if reported)	target group			
Walmsley et al. , 2019	Online open survey at baseline and at follow-up 3 months later	141	gbMSM accessing an online border-crossing guide posted by a gay men's health organization	10 months	To measure the proportion of Ontario gbMSM accessing online guides who intended to use a border-crossing approach to access PrEP an to explore their demographic characteristics, their completion of the steps in the border-crossing approach, and the barriers they perceived.	Despite high interest in pursuing an online border-crossing approach to get PrEP medications, such an approach may not be a viable option for PrEP scale-up among interested gbMSM because of logistical challenges and perceptions of safety and legitimacy.
Eccles-Radtke et al. , 2015	Descriptive report on the experience of PrEP delivery in a small PrEP clinic in Minneapolis	34	All persons at high risk of HIV acquisition (no subgroups specified)	Fall of 2012 up to June 2014	Given little real-world published data on experience with PrEP in the United States outside of a research setting, this reports wants to report experience providing PrEP through a local PrEP clinic.	Initial experience with PrEP in this setting has been favorable. No HIV seroconversions were observed among this small cohort of PrEP users. The pilot data of this program demonstrate that a majority of patients are adherent to the medication schedule when counseled about the importance of daily administration.
Liu et al. , 2019	RCT with 2:1 allocation to 'PrEPmate' versus standard of care	121	HIV-negative Young MSM (18-29 years old) at risk of HIV acquisition	9 months	To evaluate the efficacy of PrEPmate, a multi-component youth-tailored bidirectional text-messaging PrEP support intervention, in improving retention and adherence to PrEP among YMSM initiating PrEP in a safety-net clinic in Chicago.	Participants who received PrEPmate were more likely to attend study visits and to have TFV-DP levels consistent with > 4 doses per week. Overall, this multi-component mHealth intervention had a high acceptability among YMSM at risk for HIV acquisition in a real-world safety-net clinic setting.
National Department of Health, 2016	National (implementation) guidelines for expanding combination prevention and treatment options	Not applicable	All persons at high risk of HIV acquisition	Not applicable	This policy document has the goal to assist in providing the necessary guidance towards improved management of HIV prevention across different populations in the South African context, offering and promoting PrEP in the context of combination prevention.	Evidence from demonstration projects must enable the country to scale up PrEP and early HIV testing & treatment. The current platform of delivery for PrEP is through existing sex worker programmes and linkage to primary healthcare facilities, where appropriate, with the aim of integrating them into existing public health services. Additional delivery mechanisms and target populations will be incorporated in a phased approach.

Author & Year	Study Design/Study Type	Subject Under Study		Time frame	Purpose of the article	Main Findings
		n (if reported)	target group			
O' Byrne et al. , 2019	Review and case study of a nurse-led PrEP clinic	Not applicable	All persons at high risk of HIV acquisition (no subgroups specified)	Not applicable	To review the literature about PrEP and, building on USA and Canadian guidelines as well as on the author's own experience, to provide a model for how nurses can provide PrEP in STI clinics.	Nurses could enhance access to PrEP , as detailed in the author's experience with a nurse-led PrEP delivery program PrEP-RN. The authors believe such a program could be safe, effective and appropriate and could ideally target individuals most at risk of HIV infection.

PCP = Primary Care Practitioner ; MSM = Men who have Sex with Men ; TG = Transgender people ; TGW = Transgender Women ; CASI = computer-assisted self-interviewing ; CBHW = community-based health worker ; gbMSM = gay and bisexual Men who have Sex with Men ; PICME = patient-initiated continuous medical education ; AGYW = Adolescent Girls and Young Women ; CHC = Community Health Center ; HRT = Hormone Replacement Therapy ; FSW = Female Sex Workers ; DICE = drop-in center ; KP CHW = Key Population Community Health Workers
