# Supplementary material for: The current landscape of pre-exposure prophylaxis service delivery models for HIV prevention: a scoping review
Source: BMC Health Serv Res. 2020 Jul 31;20:704. doi: 10.1186/s12913-020-05568-w (PMC7395423; doi:10.1186/s12913-020-05568-w)
Supplement: Supplementary file 6 — Additional file 6. Overview of the evolution of the included literature. Overview of year of publication, geographical focus, and publication type for all included records. [file 12913_2020_5568_MOESM6_ESM.pdf]

6. Overview of the evolution of the literature.

Year	Number of articles	Geographic focus	Description of the literature
2014	1	USA	Descriptive report (n=1)
2015	1	USA	Descriptive report (n=1)
2016	2	USA, South Africa	Review (n=1) and national guidelines on PrEP (n=1)
2017	4	USA, South Africa, Kenya, Zimbabwe	Descriptive report (n=1), pilot study (n=1), a national PrEP implementation framework (n=1) and a national PrEP implementation plan (n=1)
2018	13	USA, Canada, Peru, Thailand, South Africa, Belgium, UK, New Zealand, Kenya, Australia	Descriptive report (n=7), pilot study (n=3), protocol for a pilot study (n=1), viewpoint (n=1) and a case study (n=1)
2019	12	USA, Canada, Kenya, UK, The Netherlands, France	Descriptive report (n=2), pilot study (n=4), qualitative adherence evaluation study (n=1), protocol for a RCT (n=1), national PrEP implementation report (n=1), quantitative survey (n=1), RCT (n=1) and a review (n=1)
